# Normalization of Bilateral Adrenal Gland Enlargement after Treatment for Cryptococcosis

**DOI:** 10.1155/2017/1543149

**Published:** 2017-03-26

**Authors:** Yuka Muraoka, Shintaro Iwama, Hiroshi Arima

**Affiliations:** ^1^Department of Endocrinology and Diabetes, Nagoya University Graduate School of Medicine, Nagoya 466-8550, Japan; ^2^Research Center of Health, Physical Fitness and Sports, Nagoya University, Nagoya 464-8601, Japan

## Abstract

Cryptococcosis usually occurs in immunocompromised patients and can cause enlargement of the adrenal glands, although the morphologic changes after treatment have not been reported in detail. We report the case of 24-year-old man with fevers, headaches, and impaired consciousness who had been treated with glucocorticoids for a protein-losing gastroenteropathy. The cerebrospinal fluid analysis revealed cryptococcal meningitis. Computed tomography showed bilateral adrenal enlargement. A retrospective analysis revealed that the enlargement had been detected 5 months before admission and gradually increased. The enlargement was improved with antifungal therapy and normalized 6 months later. This is the first report describing morphological changes in the adrenal glands associated with cryptococcal meningitis. Adrenal enlargement by cryptococcosis can be improved without any abnormal findings, including calcifications, which may be a unique characteristic from other diseases, including tuberculosis.

## 1. Introduction

Cryptococcosis is a fatal fungal disease caused by infections with* Cryptococcus* species. Immunocompromised patients, such as those treated with immunosuppressants (including glucocorticoids), often develop cryptococcosis [1]. Although the lungs are commonly involved in cryptococcal infections, disseminated cryptococcosis can also affect the adrenal glands. Adrenal infections with* Cryptococcus* can cause bilateral enlargement of the glands [2] but the morphologic changes after treatment have not been described in detail. Herein we report a case involving an immunocompromised patient with cryptococcal meningitis, including the morphologic findings of the adrenal glands before and after antifungal treatment.

## 2. Case Presentation

A 24-year-old man with a protein-losing gastroenteropathy due to an intestinal lymphangiectasia was treated with glucocorticoids (prednisolone, 7.5 mg/day) and developed low-grade fevers 7 months before admission. He did not have any remarkable life histories. Five months before admission, the man complained of headaches, fatigue, and a hearing abnormality. Then, he experienced nausea, diarrhea, and drowsiness for 6 days and subsequently sought evaluation at our hospital. The physical examination at the time of admission revealed that he was slow to respond (Japan Coma Scale 1-1). The following measurements were obtained: height, 161.2 cm; weight, 51.0 kg; BMI, 19.6 kg/m^2^; blood pressure, 119/78 mmHg; heart rate, 62 bpm; and body temperature, 37.4°C. The remainder of the examination findings were normal, without any signs of meningitis.

The initial laboratory data showed a white blood cell count of 11700/*μ*L, with 87.0% neutrophils (86% segmented and 1% band neutrophils), 2.0% lymphocytes, 10%monocytes, 0% eosinophils, 1% metamyelocytes, hemoglobin = 15.7 g/dL, and a platelet count of 157,000/*μ*L. The serum C-reactive protein level was slightly elevated (0.80 mg/dL). Although the serum sodium level was slightly decreased (130 mEq/L), the potassium (4.6 mEq/L), chloride (97 mEq/L), creatinine (0.59 mg/dL), fasting glucose (85 mEq/L), and HbA1c (5.1%) concentrations were normal.

**Figure 1:**
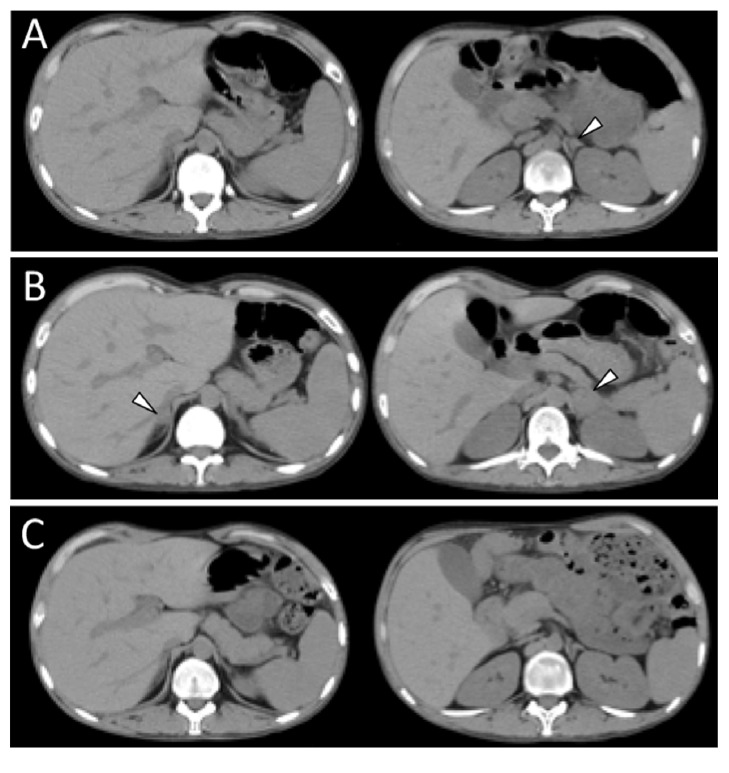
Computed tomography showing the time-course changes in the enlarged adrenal glands. Computed tomography showing gradual exacerbation of the bilaterally enlarged adrenal glands (arrowheads) evaluated at 5 months (A) and day 0 (B) before treatment and improvement at 6 months (C) after initiation of treatment.

An abdominal computed tomography (CT) showed bilateral adrenal enlargement (right, 10.0 × 20.0 mm; left, 29.0 × 29.0 mm). A retrospective analysis of the CT images revealed that the enlargement in the left adrenal gland developed 5 months before admission (Figure 1(A)), which coincided with the onset of fevers and headaches. Subsequently, the bilateral adrenal enlargement progressed (Figure 1(B)). The differential diagnosis of adrenal enlargement includes metastatic carcinoma, bilateral adrenal hyperplasia, tuberculosis, and fungal infections. A whole-body examination failed to find a primary malignant lesion. The QuantiFERON-TB test and HIV antibody titer were negative.Although there were no signs of meningeal irritation, a diagnostic lumbar puncture was performed. The cerebrospinal fluid revealed an increased white blood cell count (240/*μ*L), a normal protein level, a decreased glucose level (0.10 g/l), and a positive cryptococcal antigen titer. The pathologic specimen showed the presence of yeast-like organisms, such as* Cryptococcus* spp. on Alcian blue staining, which was subsequently determined to be* Cryptococcus neoformans*.

Although the level of serum adrenocorticotropic hormone (ACTH) was elevated (131.3 pg/mL; normal range, 7.2–63.3 pg/mL) at the time of the diagnosis of cryptococcosis (Table 1), cortisol release in response to ACTH (Cortrosyn), which was evaluated 1 day after prednisolone cessation, was increased (Table 2). Oral prednisolone (7.5 mg/day) was then resumed as treatment for the protein-losing gastroenteropathy. The other endocrinological data of adrenal gland ruled out the possibility of pheochromocytoma and aldosterone-secreting tumors in this patient (Table 2).

Amphotericin B (250 mg/day) was initiated, followed by the addition of fluconazole (400 mg/day). The symptoms improved gradually after beginning antifungal treatment. Fluconazole alone was continued after discharge. After the initiation of antifungal treatment, the elevated ACTH levels were decreased and varied during the treatment (Table 1), suggesting a stressed condition with infection at the diagnosis and unstable absorption of prednisolone due to the protein-losing gastroenteropathy. Mild hyponatremia probably due to relative adrenal insufficiency was improved to the normal range (138 mEq/L) one month after the initiation of the antifungal treatment.

An abdominal CT, which was routinely obtained during follow-up, showed that the size of the adrenal glands decreased following antifungal therapy and became normal without any abnormal findings, including calcifications, 6 months after starting treatment (Figure 1(C)).

## 3. Discussion

Cryptococcal infections usually involve the lungs and central nervous system [3] and sometimes cause disseminated lesions, including the adrenal glands [2]. Adrenal cryptococcosis, first reported in 1948 [4], often accompanies bilateral enlargement of the adrenal glands and adrenal insufficiency. In the patient presented herein, the size of adrenal glands gradually increased before treatment but decreased after antifungal therapy. The data herein suggest that the adrenal glands were infected with* Cryptococcus*. As the enlargement was detected 5 months before admission, it is possible that the adrenal infection with* Cryptococcus* was present at that time.

Although there are several reports showing enlargement of the adrenal glands in patients with cryptococcosis, the time-course changes in the morphology of the adrenal glands after antifungal treatment have not been precisely described. In most of the reports, the enlargement was mentioned [5–8] at the diagnosis or did not change, even after the antifungal treatment was effective [9–14]. There is only one report which shows a decrease in adrenal gland enlargement after treatment with amphotericin B [15]; however, the images obtained after treatment clearly showed adrenal gland enlargement. This is the first report of a dynamic change in the size of adrenal glands infected with* Cryptococcus* from the exacerbation to recovery phase. Although it is well known that adrenal tuberculosis often causes calcifications of the adrenal glands [2], the morphology of the adrenal glands in our patient improved without any abnormalities, including calcifications. While further follow-up is necessary, our case may suggest a unique feature of adrenal cryptococcosis.

There are several reports showing histopathological features of the surgical specimens or biopsy samples in adrenal cryptococcosis. The infected glands showed caseating necrosis [5, 6, 10, 13, 16] or necrotizing granuloma [6, 14], accompanied by yeast-like organism, chronic inflammation with giant cells, multinucleated histiocytes, and a few lymphocytes. Although there were no histological data from the adrenal glands in this patient, infiltration of inflammatory cells into the adrenal glands could cause the enlargement. Then, the enlargement was decreased in association with the improvement of cryptococcal infection. It is well known that calcification occurs in the inflamed lesion in tuberculosis. Although the precise mechanisms remain unclear, several possibilities have been reported, including hypercalcemia in patients with tuberculosis [17, 18] or overproduction of vitamin D from inflammatory cells [19, 20]. Given the normal serum calcium levels (4.6 mEq/L) and the improvement of the enlarged adrenal glands without calcification, it is possible that there is a different process between tuberculosis and cryptococcosis in calcification.

Despite the long-term use of prednisolone (7.5 mg/day), the basal ACTH level was elevated and cortisol release was increased in response to ACTH injection in our patient. These data suggest that (1) prednisolone administered orally was not absorbed enough to suppress the ACTH release due to the protein-losing gastroenteropathy, (2) the patient was in a stressed condition with the disease, and/or (3) he had partial adrenal insufficiency. There are several case reports which have shown the development of adrenal insufficiency in patients with adrenal cryptococcosis, especially when accompanied by meningoencephalitis [5, 6, 11, 13, 21]. It is thus important to follow adrenal function serially in our patient, although he must continue prednisolone therapy for the underlying disease. The finding that the morphology of the adrenal glands infected with* Cryptococcus* improved completely after treatment in our patient, together with the possibility that cryptococcosis can cause adrenal insufficiency, suggests that we should consider previous adrenal cryptococcosis as a possible cause of adrenal insufficiency, even if the adrenal glands are morphologically normal. In addition, since the enlargement showed slow progression, adrenal cryptococcosis may be considered as a differential diagnosis when the bilateral enlargement is present especially in immunocompromised patients. In conclusion, adrenal enlargement by* Cryptococcus* is completely reversible without any abnormality after antifungal treatment, which may be a unique characteristic from other diseases, including tuberculosis.

## Figures and Tables

**Table 1:** Endocrinological data of the patient during antifungal treatments.

	Time after initiation of antifungal treatments			
	Time of the diagnosis	2 months	4 months	6 months
ACTH (pg/mL)	131.3	4.6	28.1	1.9
Cortisol (*μ*g/dl)	38.4	16.9	14.1	6.7
Dehydroepiandrosterone-sulfate (DHEA-s) (*μ*g/dL)	482			
Epinephrine (ng/ml)	0.332	0.015		
Norepinephrine (ng/ml)	0.487	0.089		
Dopamine (ng/ml)	0.027	0.012		
Urine metanephrine (ng/mg Cr)	328	119		
Urine normetanephrine (ng/mg Cr)	63	176		
Plasma renin activity (ng/mL/hr)	3.2			
Aldosterone (pg/mL)	202.0			

**Table 2:** Rapid ACTH test.

Time	0 min	30 min	60 min
Cortisol (*μ*g/dl)	23.5	32.0	36.6
